# Reduced phosphorus is associated with older age and hypoalbuminemia. Risk factors for all-cause mortality in peritoneal dialysis patients

**DOI:** 10.3389/fnut.2023.1094256

**Published:** 2023-07-11

**Authors:** Marcela Ávila, Ma. del Carmen Prado, Miguel Ángel Cuevas-Budhart, Ramón Paniagua

**Affiliations:** Unidad de Investigación Médica en Enfermedades Nefrológicas, Hospital de Especialidades, Centro Médico Siglo XXI, Instituto del Seguro Social, Mexico City, Mexico

**Keywords:** reduced phosphorus, low serum phosphorus, all-cause mortality, cardiovascular mortality, malnutrition, peritoneal dialysis, diabetes

## Abstract

**Introduction/aim:**

Hyperphosphatemia is a mortality risk factor in dialysis patients; however, low phosphorus levels too. Diabetes and malnutrition are strongly associated with mortality and with reduced serum phosphorus. This study analyzed the pattern of serum phosphorus in patients on Peritoneal Dialysis (PD) and its association with mortality.

**Methods:**

A Secondary analysis was performed on a multicenter cohort study in peritoneal dialysis patients from two previous studies done by our group.

**Results:**

Six hundred fifty-four patients were included. Serum phosphorus was <3.6 mg/dL in 28.29% of patients, 3.6 to 5.2 mg/dL in 48.16%, and >5.2 mg/dL in 23.55%. In logistic regression analysis; education, age, and hypoalbuminemia were risk factors for low P levels. In multivariate Cox analysis *P* < 3.6 mg/dL, age, and low albumin were predictors for all-cause mortality. When lower P and lower albumin were combined, this group had the highest risk for all cause and cardiovascular mortality.

**Conclusion:**

The frequency of patients with reduced serum phosphorus was higher in the Mexican population than in Europe or Asia. Low serum phosphorus levels, older age and hypoalbuminemia were risk factors for all-cause mortality. Low phosphorus combined with low albumin levels were the highest risk factor for all-cause and cardiovascular mortality.

## 1. Introduction

The preservation of the biochemical variables of mineral metabolism is an important goal in the management of patients with chronic kidney disease (CKD) and is even more critical in patients with end-stage renal disease (ESRD) or on treatment with dialysis (1). Elevation in serum phosphorus (P) levels is one of the most frequent and best-known alterations and is the consequence of the incapacity of the damaged kidney to eliminate phosphate load from the diet (2, 3).

The elevated concentration of phosphorus has been related to a more rapid progression of renal disease (4), but even more critical, with all-cause mortality and cardiovascular mortality in particular (5, 6). Therefore, efforts to reduce intestinal absorption through the use of binders, increase clearance through dialysis, and limit secondary hyperparathyroidism, have been the source of abundant literature in nephrology (7).

The percentage of patients treated with dialysis who have serum phosphorus values over the standard upper limit (5.5 mg/dL) is greater than 50%, but the frequency of patients with values under the standard lower limit (3.5 mg/dL) (8), is also important, with up to 10% in Europe and some studies report that 19.7% of patients are in the lowest quintile (<4.4 mg/dL) (9–12).

There is a perception that hyperphosphatemia is less frequent in the Mexican population, given that some studies related to mineral metabolism have reported low prevalence in this country (13, 14). Among the causes that may explain these findings is the higher frequency of diabetes, malnutrition, and inflammation, conditions where phosphorus is usually reduced; these conditions are more frequent in our environment compared with other countries (15). It should also be noted that reduced preferences and less availability of food with high phosphorus content and reduced financial ability to purchase them are factors associated with patient social and cultural level in developing countries.

The low phosphorus level is relevant since phosphorus is essential for life; it is actively involved in many critical biochemical pathways, such as energy and nucleic acid metabolism, cellular signaling, and bone formation. It is a crucial component of cellular membrane phospholipids, nucleic acids, adenosine triphosphate (ATP), and phosphoproteins.

The role of hypophosphatemia on the clinical outcomes of the dialysis population is controversial. It is associated with mortality, but the finding is not consistent. However, the association remains statistically significant after adjustments for markers of malnutrition-inflammation-cachexia syndrome. The association of hypophosphatemia with other clinical conditions, such as cardiovascular dysfunction, metabolic syndrome, or glucose intolerance found in the non-CKD population, has not been analyzed in dialysis patient (16).

Recognized cardiovascular risk factors, such as diabetes, malnutrition, inflammation, low economic and cultural levels, are frequent findings in the Mexican population; these same factors are associated with low blood concentrations of phosphorus. For this reason, hyperphosphatemia and hypophosphatemia are equality relevant as a cardiovascular risk factor. This study aimed to analyze the distribution pattern of serum phosphorus in the Mexican population with peritoneal dialysis and its association with general and cardiovascular mortality.

## 2. Materials and methods

### 2.1. Design

Secondary analysis was performed on a multicenter cohort study of incident patients on peritoneal dialysis of a two previous studies done by our group, the first (study A) was a non-intervention study to analyze adherence to clinical practice guidelines in patients on dialysis, conducted between 2007 and 2009 (13), and the second (study B) was a study without intervention to find out the frequency of complications in incident patients on PD between 2015 and 2017 (17), both were from multicenter cohorts. Followed up by 18 months.

### 2.2. Population

Data from patients were included if they were adults (>18 years) and free of acute complications for at least 1 month before inclusion in the study; there was no restriction by gender or cause of renal disease. All patients initiated peritoneal dialysis in a planned manner.

Exclusion criteria: patients were serologically positive for hepatitis B or C or HIV, had a recent renal transplant, or were under treatment with immunosuppressant.

#### 2.2.1. Primary outcomes

Mortality from any cause and cardiovascular mortality are defined by the following events: acute myocardial infarction, heart failure, brain-vascular disease, peripheral vascular disease, arrhythmia, and sudden death.

#### 2.2.2. Data collection

Demographic data and history of renal disease were obtained from clinical files by nurses trained in clinical research and registered on pre-established forms. Data gathered included: age, gender, and diagnosis of diabetes. Patients received conventional treatment with dextrose solutions, and during scheduled visits, weight, height, and body mass index were recorded. Patients were followed for at least 18 months from the inclusion of the last patient and were censored at the date of death, transfer to hemodialysis or transplant, or loss to follow-up.

Laboratory Biochemical analyses were performed in a central laboratory with standard automated techniques. Measurements included: phosphorus, total calcium, total calcium corrected for albumin, C-reactive protein (CRP), glucose, urea, and creatinine. Samples were preserved at −70°C until analysis. For the ends of the analysis, only baseline data were considered. The variables related with mineral metabolism were classified as follows: calcium corrected for albumin (cCa), Q1: <8.4; Q2 to Q3: >8.4–9.5; Q4 >9.5 mg/dL; serum phosphorus in quartiles; Q1: <3.6 mg/dL, Q2 to Q3: 3.6–5.2 mg/dL, Q4: >5.2 mg/dL. Parathyroid hormone (PTH); low <150; in range >150 to <300; high >300 ng/mL).

### 2.3. Statistical analysis

Data are presented as mean ± standard deviation for continuous variables and percentages for discrete variables. For comparisons between groups, one-way ANOVA or Student’s t was used for continuous variables and X^2^ for discrete variables. The classification criteria were according to the quartiles of the concentration of phosphorus.

Logistic regression was used to analyze the risk factors for having low phosphorus concentrations, and Cox proportional risk model was used to analyze mortality. We did the competing risk models analysis for cardiovascular mortality. Significant variables in univariate analysis were included in multivariate analysis using the step-forward method. All statistical tests were done with the program Statistical Package for Social Sciences (SPSS-PC), v.24 (SPSS, Chicago, IL, USA), and Statistics/Data Analysis (STATA), v 14.2.

## 3. Results

### 3.1. Baseline data

Table 1 shows baseline results of demography, non-medical and medical variables when patients were classified according to the quartiles of serum phosphorus. The analysis included 654 patients. The percentage of patients in quartile 1, Q1 (<3.6 mg/dL) was 28.3%. Regarding non-medical variables, patients with low phosphorus were older, mostly women, with low education levels, and had home and employee activity. They tended to have lower economic income (not significant). Regarding medical variables, the low phosphorus group had a higher percentage of diabetes, lower diastolic pressure, lower concentrations of urea, creatinine, albumin, cCa, PTH, and CRP, and higher levels of glucose, triglycerides, and residual renal function (RRF). Continuous Ambulatory Peritoneal Dialysis (CAPD) was the dialysis modality in 525 patients (80.3%), and Automated Peritoneal Dialysis (APD) in 129 patients (19.7%).

**TABLE 1:** Demography, non-medical, and medical variables classified by phosphorus quartiles.

	Total	Q1 (< 3.6 mg/dL)	Q2 (3.6–4.2 mg/dL)	Q3 (4.3–5.1 mg/dL)	Q4 (>5.2 mg/dL)	*p*
*n* (%)	654 (100)	185 (28.29)	146 (22.32)	169 (25.84)	154 (23.55)	
Age (year)	48.32 ± 15.1	54.12 ± 12.5^a^	51.68 ± 13.8^b^	47.29 ± 15.2	39.30 ± 14.7^c^	0.001
Gender, *n* (%)						0.035
Male	267 (40.8)	91 (34.1)	59 (22.1)	64 (24.0)	53 (19.9)	
Female	387 (59.2)	94 (24.3)^b^	87 (22.1)	105 (24.0)	101 (26.1)	
Economic income, *n* (%)						0.190
Low	373	107 (23.4)	87 (19)	102 (2.3)	77 (16.8)	
Medium-high	84	32 (7)	19 (4)	23 (5)	10 (2.2)	
Education, *n* (%)						0.001
Low	199	80 (17.5)^a^	49 (10.7)	46 (10.1)	24 (5.2)^d^	
Intermediate	173	38 (8.3)	47 (10.3)	49 (10.7)	39 (8.5)	
High	85	21 (4.6)	10 (2.2)	30 (6.6)	24 (5.2)	
Activity, *n* (%)						0.001
Non or home	155	38 (8.3)	37 (8.1)	45 (9.8)	35 (7.7)	
Employee	274	101 (22.1)^a^	66 (14.4)	68 (14.7)	39 (8.5)^d^	
Professional	28	0	30 (0.7)	12 (2.6)	13 (2.6)	
Diabetes mellitus						0.001
Yes *n* (%)	357	125 (28.7)	93 (21.4)	99 (22.7)	59 (13.6)	
Weight (k)	65.53 ± 13.43	64.85 ± 11.87	66.16 ± 13.35	64.70 ± 13.76	66.56 ± 14.83	0.474
Height (cm)	160.21 ± 10.20	158.49 ± 8.91^b^	160.33 ± 10.95	160.34 ± 11.77	162.03 ± 8.70	0.016
BMI (k/m^2^)	25.68 ± 7.23	25.79 ± 4.09	26.06 ± 9.05	25.57 ± 9.60	25.29 ± 4.87	0.818
SBP (mmHg)	131.83 ± 22.95	134.17 ± 23.14	132.15 ± 22.77	129.88 ± 21.64	130.86 ± 24.24	0.330
DBP (mmHg)	80.82 ± 14.08	79.18 ± 14.00^b^	81.40 ± 13.37	79.91 ± 14.32	83.25 ± 14.33^d^	0.045
sGlucose (mg/dL)	130.19 ± 81.51	145.85 ± 100.25^a^	143.93 ± 93.75	120.99 ± 65.61	108.46 ± 46.69	0.001
Urea (mg/dL)	102.91 ± 39.11	84.44 ± 33.81^a^	92.99 ± 30.39^b^	106.10 ± 35.33	130.99 ± 39.97^c^	0.001
Creatinine (mg/dL)	7.74 ± 3.42	6.14 ± 2.82^a^	6.47 ± 2.74	7.72 ± 2.76	10.87 ± 3.25	0.001
Cholesterol (mg/dL)	179.45 ± 42.62	175.21 ± 44.87	179.11 ± 41.61	181.54 ± 41.57	182.53 ± 41.93	0.386
HDL Cholesterol (mg/dL)	38.53 ± 20.27	39.81 ± 20.07	37.35 ± 15.37	40.35 ± 26.97	35.32 ± 13.29	0.254
Triglycerides (mg/dL)	177.53 ± 101.97	197.32 ± 142.05^e^	176.05 ± 87.99	165.00 ± 78.59	168.94 ± 72.72	0.014
sAlbumin (g/dL)	3.23 ± 0.56	3.02 ± 0.54^a^	3.20 ± 0.56^b^	3.23 ± 0.52^c^	3.50 ± 0.53^d^	0.001
CRP (mg/L)	5.23 ± 9.06	6.11 ± 10.00	5.48 ± 8.31	4.39 ± 8.6	4.9 ± 8.2	<0.315
Phosphorus (mg/dL)	4.43 ± 1.32	3.03 ± 0.52^a^	3.93 ± 0.17^b^	4.66 ± 0.26^e^	6.31 ± 0.92^c^	0.001
tCa (mg/dL)	8.71 ± 1.33	8.19 ± 1.62^a^	8.86 ± 1.14	8.84 ± 0.96	9.05 ± 1.29^d^	0.001
cCaAlb (mg/dL)	9.33 ± 1.24	8.97 ± 1.53^a^	9.50 ± 1.08	9.45 ± 0.91	9.45 ± 1.25^d^	0.001
PTH (pg/mL)	34.63 ± 66.98	27.06 ± 51.70	20.27 ± 34.04	40.31 ± 71.74	53.98 ± 96.84	0.004
RR F (mL/min)	2.85 ± 2.0	3.28 ± 2.42	3.33 ± 2.35^b^	2.76 ± 2–12^c^	1.77 ± 1.33^d^	0.001
Ultrafiltration (mL/24 h)	837.82 ± 445.73	798.24 ± 449.43	861.11 ± 430.94	913.22 ± 483.49	763.90 ± 385.45	0.061
Follow-up (months)	17.72 ± 7.79	16.63 ± 8.07	17.43 ± 6.88	18.48 ± 8.16	18.46 ± 7.73	0.078
Deaths, all-cause (n)	93	36	14	21	22	0.066
Death rate (events/100 pts/year)	9.7	3.9	1.5	2.1	2.2	
Deaths, cardiovascular (n)	54	17	7	15	16	0.383
Death rate, cardiovascular (events/100 pts/year)	5.6	1.9	0.7	1.5	1.6	

Data are expressed as mean ± SD, or in frequency (%). SBP, systolic blood pressure; DBP, diastolic blood pressure; CRP, C–reactive protein; cCa, albumin-corrected calcium; PTH, parathyroid hormone; RRF, residual renal function = the mean creatinine and urea clearance. One-way ANOVA test with the significance of *p* < 0.005, *p* < 0.05, test *post-hoc*: ^a^Q1 vs. Q3, Q4. ^b^Q2 vs. Q3, Q4. ^c^Q4 vs. Q3. ^d^Q1 vs. Q4. ^e^Q1 vs. Q3.

### 3.2. Follow-up

During the follow-up, 93 patients died (14.2%), 24 patients (3.7%) were transferred to hemodialysis, 22 patients (3.4%) were transplanted and 41 patients (6.3%), were lost at the end of the follow-up (Change of address, transfer to other hospitals, loss of insurance rights, voluntary withdrawal).

The causes of Death were: Cardiovascular Death in 54 patients (58%) (which include acute myocardial infarction in 22 patients (23.6%), heart failure in 7 (7.5%), arrhythmia in 6 (6.4%), stroke in 7 patients (7.6%), sudden Death in 10 patients (10.8%), other cardiovascular causes in 2 patients, (2.1%), PD-related peritonitis in 8 patients (8.6%), infections (except peritonitis) in 11 patients (11.8%), uremia/hyperkalemia/acidosis in 13 patients (14%), unknown in 7 patients (7.6%).

**FIGURE 1:**
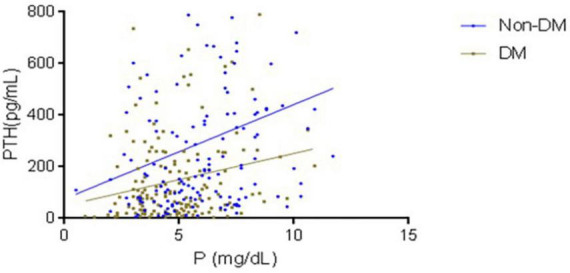
The regression line between parathyroid hormone and phosphorus levels in non-diabetic and diabetic patients. PTH was lower in diabetic than non-diabetic patients, at the same phosphorus concentrations. The regression equation for DM was: *y* = 6.84(x) + 4.05, and for non-DM: was *y* = 10.1(x) + 8.34.

Figure 1 shows the regression line between Parathyroid Hormone and Phosphorus levels in Non-diabetic (nDM) and Diabetic Patients (DM). PTH was lower in Diabetic than non-diabetic patients at the same phosphorus concentrations. The regression equation for non-DM: was *y* = 10.1(x) + 8.34, and for DM was: *y* = 6.84(x) + 4.05. DM have lower slop and intercept.

Table 2 shows logistic regression analysis to know the relative value of non-medical risk factor for low serum phosphorus, in Model 1, low education was significant. In model 2, was Model 1 plus; diabetes, serum albumin and age. Albumin and age were the factors most related to low phosphorus.

**TABLE 2:** Effect of non-medical plus medical variables on low phosphorus levels.

	Variable	Significance p	RR	95% CI	
Model 1	Education level	0.000			
	Elementary	0.006	2.26	1.26	4.05
	High School	0.865	0.95	0.508	1.76
	Economic income				
	Low	0.053	0.60	0.360	1.07
	Medium, high (reference)				
Model 2	Education level	0.130			
	Elementary	0.128	1.64	0.87	3.11
	High School	0.960	1.02	0.53	1.95
	Economic income				
	Low	0.147	0.62	0.364	1.04
	Medium, high (reference)				
	Diabetes	0.125	1.51	0.89	2.55
	Albumin (g/dL)	0.001	0.49	0.32	0.76
	Age	0.011	1.03	1.00	1.04

Model 1 Logistic regression analysis with education level and economic income. Model 2; Model 1 plus diabetes, albumin and age.

**FIGURE 2:**
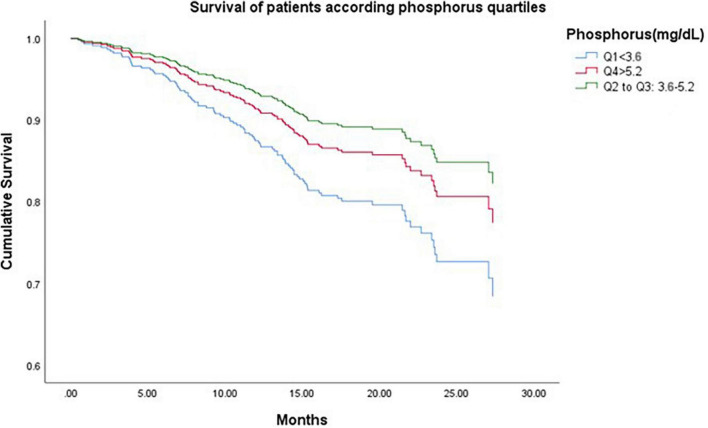
All-cause survival according to phosphorus quartiles (Cox analysis). Blue line; Q1: <3.6 mg/dL; red line; Q4 >5.2 mg/dl, and green line, Q2 to Q3 = 3.6 to 5.2 mg/dL. The patients in Q2 to Q3 had the best survival, followed by Q4 and Q1, respectively, taking the value of Q2–Q3 as a reference. Q1: HR 1.94 (95% IC 1.21–3.1, *p* < 0.006), Q4: HR 1.38 (95% IC 0.770–2.22, *p* < 0.32).

Figure 2 shows all-cause survival according to phosphorus quartiles (Cox analysis). Blue line; Q1 < 3.6 mg/dL; red line; Q4: >5.2 mg/dl, and green line, Q2-Q3: 3.6 to 5.2 mg/dL of phosphorus. The patients in Q2-Q3 had the best survival, followed by Q4 and Q1, respectively, taking the value of Q2–Q3 as a reference. That is, the phosphorus concentrations, concerning mortality risk, have a U shape. Both low and higher serum phosphorus levels were associated with elevated mortality risk.

Table 3 shows the multivariate Cox regression analysis of factors associated with all-cause mortality.

**TABLE 3:** Factors associated with all-cause mortality on peritoneal dialysis patients.

Variable	Significance p	HR	95% CI	
P Clasification	0.002			
Q2–Q3	0.147	0.69	0.425	1.13
Q1	0.032	1.89	1.05	3.40
Ca Clasification	0.407			
Q2–Q3	0.232	1.35	0.82	2.24
Q1	0.321	1.29	0.78	2.13
Age (year)	0.001	1.05	1.02	1.07
Diabetes (no)	0.090	0.61	0.34	1.08
Albumin (g/dL)	0.001	0.42	0.28	0.64

Multivariate analysis (adjusted Cox analysis) of factors associated with all- cause mortality. Significance = *p* < 0.05, *p* < 0.005.

Phosphorus in quartile 1 (QI) < 3.6 mg/dL (HR: 1.89; 95% CI; 1.05–3.4, *p* < 0.032) as a risk, older age (HR: 1.05, 95% CI; 1.02–1.07, *p* < 0.001) and low albumin concentrations (HR: 0.42; 95% CI 0.28–0.64, *p* < 0.001), as a protector, were associated with elevated all-cause mortality risk, while Q2–Q3 (3.5–5.2 mg/dL), calcium and diabetes had no effect. Taking as a reference Q4. Neither level of phosphorus nor calcium had a significant effect on cardiovascular mortality.

To clarify the role of low phosphate as an independent risk factor, we classified serum Albumin (Alb), and phosphorus (P) levels, where the cut-off point was the low quintile value. We made four categories of patients; Group 1 (*n* = 44): low Alb and low P; Group 2 (*n* = 85) low Alb-high P; Group 3 (*n* = 96): high Alb-and low P and Group 4 (*n* = 425), high Alb and high P. Further comparisons between the subgroups were analyzed using Cox regression models. in all-cause and cardiovascular mortality.

**FIGURE 3:**
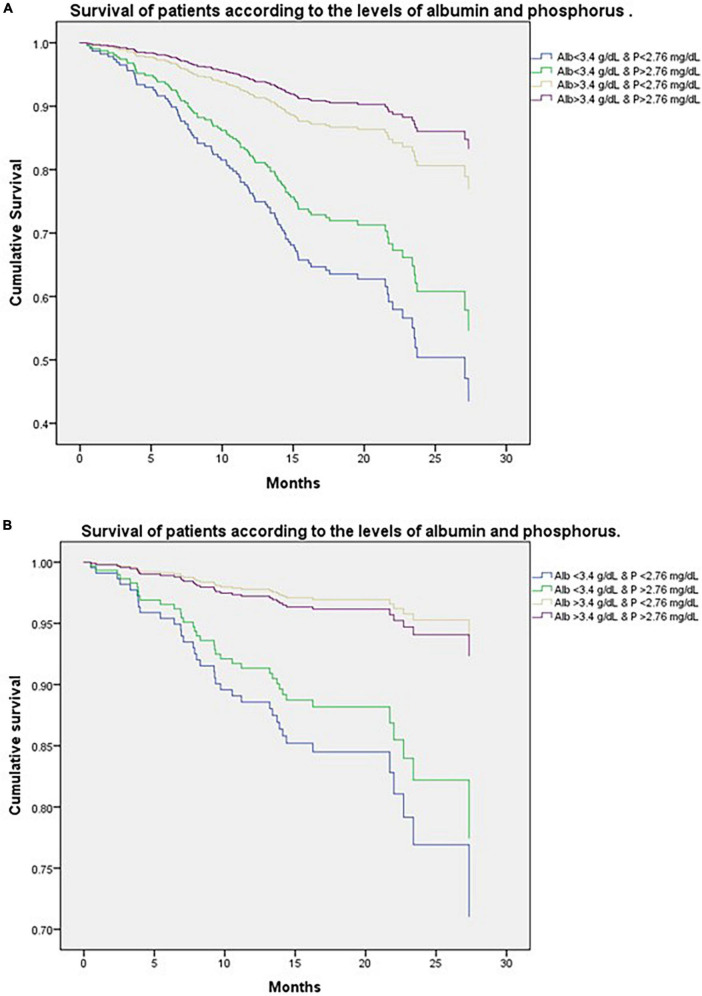
**(A)** Survival curves of patients classified according to the combination of albumin (Alb) and phosphorus (P) levels, analyzed using Cox models for all-cause mortality. The group of patients with low Alb and low P levels had the lowest risk of survival, followed by the group with low Alb and high P, followed by the group with Alb high and P low and Alb high and P high, respectively. **(B)** Survival of patients classified according to the combination of albumin (Alb) and phosphorus (P) levels, analyzed using Cox models for cardiovascular mortality. The group of patients with low Alb and low P levels had the lowest risk of survival, followed by the group with low Alb and high P, followed by the group with Alb high and P high, and then the group with Alb high and P low, respectively.

Figure 3A shows the survival curves to know the all-cause mortality of patients classified according to the combination of Alb and P levels.

The group of patients with low Alb and low P levels had the highest risk of all-cause mortality, with a HR: 2.9 (95% CI; 1.854.7, *p* < 0.001) followed by the group with low Alb and high P, HR: 2.20 (95% CI; 1.53–3.1, *p* < 0.005), followed by the group high Alb and low P, there was not difference between this group and high Alb and high P, HR: 1.34 (95% CI; 0.92–1.9, *p* < 0.128).

This means that the association between phosphorus concentration and mortality was modified by albumin level.

Figure 3B shows the survival curves to know the cardiovascular mortality of patients classified according to the combination of Alb and P levels, analyzed using Cox models.

The group of patients with low Alb and low P levels had the highest risk of cardiovascular mortality, with HR: 4.29 (95% CI; 1.69–10.4, *p* < 0.002) followed by the group with low Alb and high P; HR: 3.21 (95% CI; 1.43–7.18, *p* < 0.005), the group with the longest survival, but not significant was the group with Alb high and P low, with HR: 0.79 (95% CI; 0.23–2.69, *p* < 0.70).

These results show that low and high phosphorus levels in conjunction with low albumin values are the highest risk of death. The competing risk models analysis for cardiovascular death, shows that competing variables (change to hemodialysis, transplantation, change of address, transfer to other hospitals, loss of insurance right, voluntary withdrawal, and all-cause of mortality), not affected to cardiovascular death, it was: Q2-Q3: HR; 0.66 (95% CI 0.35–1.2, *p* < 0.216), Q4; HR: 0.69 (95% CI 0.425–1.13, *p* < 0.47), compared with Q1. For all-cause mortality, Q2-Q3: HR 0.51 (95% CI 0.32–0.82, *p* < 0.05) y Q4: HR 0.66 (95% CI 0.39–1.13, *p* < 0.191). Competing variables do not affect the mortality.

## 4. Discussion

The results reported in this study support the clinical perception that the proportion of patients with serum phosphorus levels lower than those recommended in clinical guideless practices is greater in Mexico than in other countries. Non-medical factors such as low education and non-professional activity are associated with low phosphorus. Medical factors such as serum markers of malnutrition and inflammation had closer associations with low phosphorus than non-medical ones. In multivariate analysis, low phosphorus levels older age, and albumin impacted all-cause mortality. But the combination of low phosphorus and low albumin levels was the strongest risk for cardiovascular and all-cause death.

Preservation of phosphorus concentrations within the accepted targets of the clinical practice guidelines has been a cause of concern in treating patients with CKD-ESRD. Nevertheless, such limits vary among the most widely distributed guidelines; even more, there are also significant variations in regional or national guidelines (9).

Concentrations of phosphorus in patients with CKD-ESRD are generally over the recommended limits; in the first reports of the Dialysis Outcomes and Practice Patients Study (DOPPS), 51.6% of the patients had >5.5 mg/dL and individual values by country were 53.6, 49.4 and 51.9% in Japan, Europe, and the USA, respectively, with a trend toward an increase in the percentage of patients within targets as reported in later studies.

In this study, the percentage of patients with hyperphosphatemia was lower, (23.5%) than in the studies mentioned above. The same DOPPS studies showed that 7.6% of the total population had phosphorus concentrations lower than the recommended limits (3.5 mg/dL). The percentage of patients with low phosphorus varied in the various countries studied, with 5.8% in Japan, 10.1% in Europe, and 6.8% in the United States (10, 11). The percentage of patients with phosphorus below 3.6 mg/dL found in this study was (28.3%) substantially higher when compared with the reports above. It confirms the clinical perception of low phosphorus in Mexican patients.

The causes of low phosphorus have not been well established although it appears to be related to medical and non-medical factors. Among the non-medical ones involved are economic and cultural factors. Population with low socio-economic level are more susceptible to have low phosphorus levels and have a faster progress of CKD (18), later access to treatment, and to be malnourished (19, 20). They more frequently consume unprocessed, rather than processed foods because in México they are cheaper. Processed foods contain much additives and preservatives, which have a high phosphorus content. Our results show that the group of patients with phosphorus <3.6 mg/dL had significant proportions of low income and education, factors that imply higher consumption of unprocessed foods, malnutrition, inflammation and low albumin levels (21, 22). Insufficiency of protein intake may also result in lower serum phosphorus concentration and a concurrent decline in serum albumin.

In patients on peritoneal dialysis, the total removal of phosphorus depends more on residual renal function than on peritoneal extraction (23). So in patients with poor renal function, such as in this study, peritoneal dialysis has little effect on phosphorus balance. The reduced food intake plays a fundamental role, and uremia *per se* is associated with spontaneous reduction of food intake (24, 25), a reduction that is even more critical in patients with the malnutrition-inflammation syndrome (26).

In this study, neither appetite nor phosphorus intake was explicitly measured, but the association with malnutrition-inflammation can be seen in the narrow correlation between serum phosphorus, serum albumin, and CRP, Table 1, which are markers of this syndrome (27).

With respect to phosphate binders, CaCO_3_ the most frequently used. There was no significant difference in serum phosphorus levels between those who ingested CaCO_3_ and not. We believe that it was due to the fact that the average consumption was small; 1.6 g/day/kg.

The importance of the medical factors surpassed the non-medical ones, as seen in logic regression analysis, where these latter maintained independent values as factors associated with low phosphorus levels.

Among the medical factors, one should consider inadequate elimination and insufficient phosphorus ingestion. Limited studies in CKD animal models and patients with CKD suggest that there may be a break in this homeostatic response where the intestine fails to compensate for impaired renal phosphorus excretion by reducing fractional intestinal phosphorus absorption (28). Also reduced levels of 25-hidroxy vitamin D in CKD patients may impair phosphorus absorption.

The value of hyperphosphatemia as a risk factor for all-cause and cardiovascular mortality has been previously found (2, 4–6). However, the association between serum phosphorus and mortality is not linear; various studies have shown a U-shaped association with greater risk at both ends, (29) but mortality has different characteristics. While high phosphorus levels have been related to all-cause and cardiovascular death, low levels have been associated with malnutrition-inflammation and, in consequence, death by infections and all-cause mortality (30). In our study, this situation was demonstrated clearly in Figure 2. Mortality was higher in Q1 and Q4 than in Q2–Q3.

The present study, the univariate analysis showed the association between low phosphorus and all-cause mortality, but not with cardiovascular mortality. In multivariate analysis, conditions such as age, and hypoalbuminemia were independent risk factors for mortality with greater significance than phosphorus reduction, Table 3. With the competition analysis we were able to corroborate that the competition variables, such as losses (change to hemodialysis, transplantation, change of address, transfer to other hospitals, loss of insurance rights, voluntary withdrawal) of patients from the study and all-cause mortality, did not affect cardiovascular death.

We wanted to know the mortality risk of phosphorus in combination with albumin, and we found that the association between phosphorus concentration and mortality was modified by albumin level. It is important to know that low phosphorus and low albumin were risk factors for all-cause and cardiovascular mortality, but low phosphorus with high albumin blunted these effects of the low phosphorus on all-cause and cardiovascular mortality, such in others studies (31).

Low phosphorus concentrations added to malnutrition significantly increase the risk of death in vulnerable populations, such as geriatric patients (32).

Our study has strengths and weaknesses. The strength lies in a sample size considered large from a multi-center cohort with and selection criteria allowed us to analyze a representative sample of a common population of PD patients in México. The main weakness is that this is a secondary study without intervention on the supply or elimination of phosphorus in urine or dialysis. However, there are currently no controlled clinical studies that show that lowering P leads to better survival.

The data in this study do not contradict the predictive value or the importance of hyperphosphatemia in all-cause or cardiovascular mortality. They simply warm of the clinical importance of low phosphorus levels and their relation with the diabetic state, malnutrition, inflammation, and the effect of socio-economic factors as impulses for this dangerous relation for the life of patients.

In conclusion, the present study showed that the frequency of patients with serum phosphorus less than 3.6 mg/dL was higher in the Mexican population than in Europe or Asia; it also shows that low education level, older age, women, and less productive activities were risk factors for low serum phosphorus. Reduced serum phosphorus levels, older age and hypoalbuminemia were risk factors for all-cause mortality. But low phosphorus combined with low albumin values were the highest risk for all-cause mortality and cardiovascular mortality in peritoneal dialysis patients.

## Data availability statement

The raw data supporting the conclusions of this article will be made available by the authors, without undue reservation.

## Ethics statement

The original protocol was approved by the Comité Nacional de Investigación Científica, and the Comité de Bioética from Instituto Mexicano del Seguro Social (IMSS). Registration number, R-2016-785-058. The patients/participants provided their written informed consent to participate in this study.

## Author contributions

MÁ and RP: conceptualization, formal analysis, and writing review and editing. MP: methodology. MÁ and MP: investigation. MC-B: data curation. MÁ and MC-B: writing and original draft preparation. RP: funding acquisition. All authors have read and agreed to the published version of the manuscript.
